# Brain Metabolism Alterations Induced by Pregnancy Swimming Decreases Neurological Impairments Following Neonatal Hypoxia-Ischemia in Very Immature Rats

**DOI:** 10.3389/fneur.2018.00480

**Published:** 2018-06-25

**Authors:** Eduardo F. Sanches, Yohan Van de Looij, Audrey Toulotte, Analina R. da Silva, Jacqueline Romero, Stephane V. Sizonenko

**Affiliations:** ^1^Division of Child Development and Growth, Department of Pediatrics, University of Geneva, Geneva, Switzerland; ^2^Laboratory for Functional and Metabolic Imaging, Ecole Polytechnique Fédérale de Lausanne, Lausanne, Switzerland

**Keywords:** prematurity, hypoxia-ischemia, pregnancy swimming, neuroprotection, magnetic resonance imaging, brain

## Abstract

**Introduction:** Prematurity, through brain injury and altered development is a major cause of neurological impairments and can result in motor, cognitive and behavioral deficits later in life. Presently, there are no well-established effective therapies for preterm brain injury and the search for new strategies is needed. Intra-uterine environment plays a decisive role in brain maturation and interventions using the gestational window have been shown to influence long-term health in the offspring. In this study, we investigated whether pregnancy swimming can prevent the neurochemical metabolic alterations and damage that result from postnatal hypoxic-ischemic brain injury (HI) in very immature rats.

**Methods:** Female pregnant Wistar rats were divided into swimming (SW) or sedentary (SE) groups. Following a period of adaptation before mating, swimming was performed during the entire gestation. At postnatal day (PND3), rat pups from SW and SE dams had right common carotid artery occluded, followed by systemic hypoxia. At PND4 (24 h after HI), the early neurochemical profile was measured by ^1^H-magnetic resonance spectroscopy. Astrogliosis, apoptosis and neurotrophins protein expression were assessed in the cortex and hippocampus. From PND45, behavioral testing was performed. Diffusion tensor imaging and neurite orientation dispersion and density imaging were used to evaluate brain microstructure and the levels of proteins were quantified.

**Results:** Pregnancy swimming was able to prevent early metabolic changes induced by HI preserving the energetic balance, decreasing apoptotic cell death and astrogliosis as well as maintaining the levels of neurotrophins. At adult age, swimming preserved brain microstructure and improved the performance in the behavioral tests.

**Conclusion:** Our study points out that swimming during gestation in rats could prevent prematurity related brain damage in progeny with high translational potential and possibly interesting cost-benefits.

**HIGHLIGHTS**

- Prematurity is a major cause of neurodevelopmental impairments;
- Swimming during pregnancy reduces brain damage after HI injury;
- Pregnancy is an important but underestimated preventive window.

## Introduction

### Prematurity and neonatal hypoxia-ischemia

Preterm birth represents around 11% of all live births (~15 million children) (1) and is one of the most important causes of perinatal mortality and morbidity. Despite the progress of neonatal medicine improving their survival rate, the incidence of premature babies has increased in most of the countries (2–4). Prematurity is linked to subcortical white and gray matter lesions and to impaired structural connectivity (5, 6), leading to lifelong neurodevelopmental disturbances (7–10).

Neonatal hypoxic-ischemic (HI) brain injury is a major public health problem leading to complications during and after birth (11), and is part of the etiology of cerebral palsy, neurodevelopmental deficits, learning disabilities, ADHD, autism and other diseases (4, 12). HI leads to a distinct neurological injury pattern depending at gestational age it occurs. In preterm infants, brain injury leads to a diffuse pattern of white matter damage with altered myelination, ventriculomegaly and reduced cortical development or to cystic periventricular leukomalacia whereas in full-term newborns, the gray matter areas are the primary regions injured (12). HI occurs due to a drop in the brain blood and/or oxygen flow (13) which compromises the oxidative metabolism, leading to a decrease in energy levels and increased glutamate release, leading to excito-oxidative injury cascade (11), metabolic failure, alterations in the neuron-glia coupling and cell death (14). Multimodal magnetic resonance techniques can be used to monitor metabolic and microstructural changes following HI (15). Localized ^1^H-Magnetic resonance spectroscopy (MRS) has been used to follow biochemical changes in the pup rat brain following HI (16). In addition, diffusion tensor imaging (DTI) probes the brain microstructure and allows evaluation of microstructural alterations in the brain following HI (17).

The Rice-Vannucci rodent model is often used to mimic the pathological mechanisms as well as the functional consequences of hypoxia-ischemia, allowing a better comprehension of the HI pathophysiology and evaluating effects of therapeutic strategies [for a review, see 18]. In terms of cerebral maturity, the 3-day-old rat corresponds to a preterm human baby birth at 24–28 weeks of gestation and is used to study the mechanisms of perinatal brain damage in this population defined as early preterm (19–24). HI in early preterm leads to disruption in cell development and in the cortical cytoarchitecture (23), inflammation, alterations in myelination and cognitive impairments (21, 24, 25). HI pathophysiology complexity enables multiple therapeutic targets and neuroprotective strategies can counteract one or, ideally, multiple pathways (26–28).

### Physical exercise benefits during pregnancy

The importance of gestational interventions that improve maternal, perinatal, and neonatal health outcomes is recognized (10, 29). Exercise during pregnancy is considered beneficial to both mother and fetus and is recommended by the Colleges of Obstetricians and Gynecologists (30, 31). Several risk factors such as diabetes mellitus (32) and preeclampsia, commonly associated to premature delivery (33, 34) can be reduced by physical exercise (35, 36). Preclinical studies evidenced that pups born from exercised mothers had significantly higher brain, liver, heart and kidney weights compared to the controls, which suggests that regular exercise during pregnancy can improve placentary functioning and support fetal development (37, 38). Labonte-Lemoyne et al. suggested that babies born from exercised mothers were born with more mature brains (39).

Swimming during pregnancy is widely recommended for women (40) due to the low-impact effects of buoyancy (41), the excellent heat conductor capacity (42) and the beneficial effects on the cardiovascular system in the mother (40), and although some evidence demonstrates that urinary tract infections (UTI) could eventually occur, which could imply birth defects, literature shows no significant association between swimming during pregnancy and UTI (43). Besides, preclinical evidence show that pregnancy swimming can improve the intrauterine environment and improve brain maturation in the pups (38, 44, 45) by inducing hippocampal neurogenesis (46, 47, 48), enhancing the brain antioxidant capacity (49), maintaining the ionic gradients as well as the levels of neurotrophins (38, 46, 50) and leading to an improvement in cognitive tasks (38, 48, 50). Although pregnancy swimming could have a positive impact over multiple pathways involved in HI injury, the knowledge about its potential beneficial effects are still limited. Thus, we hypothesize that pregnancy swimming can induce metabolic adaptations in the pup's brain that are sufficient to reduce a subsequent HI damage. Using a multimodal approach involving *in vivo* MR spectroscopy and *ex vivo* MR imaging techniques, biological and behavioral evaluation we assessed the potential neuroprotective effects of gestational swimming on HI brain injury in the immature rat brain.

## Materials and methods

### Animals

**Figure 1:**
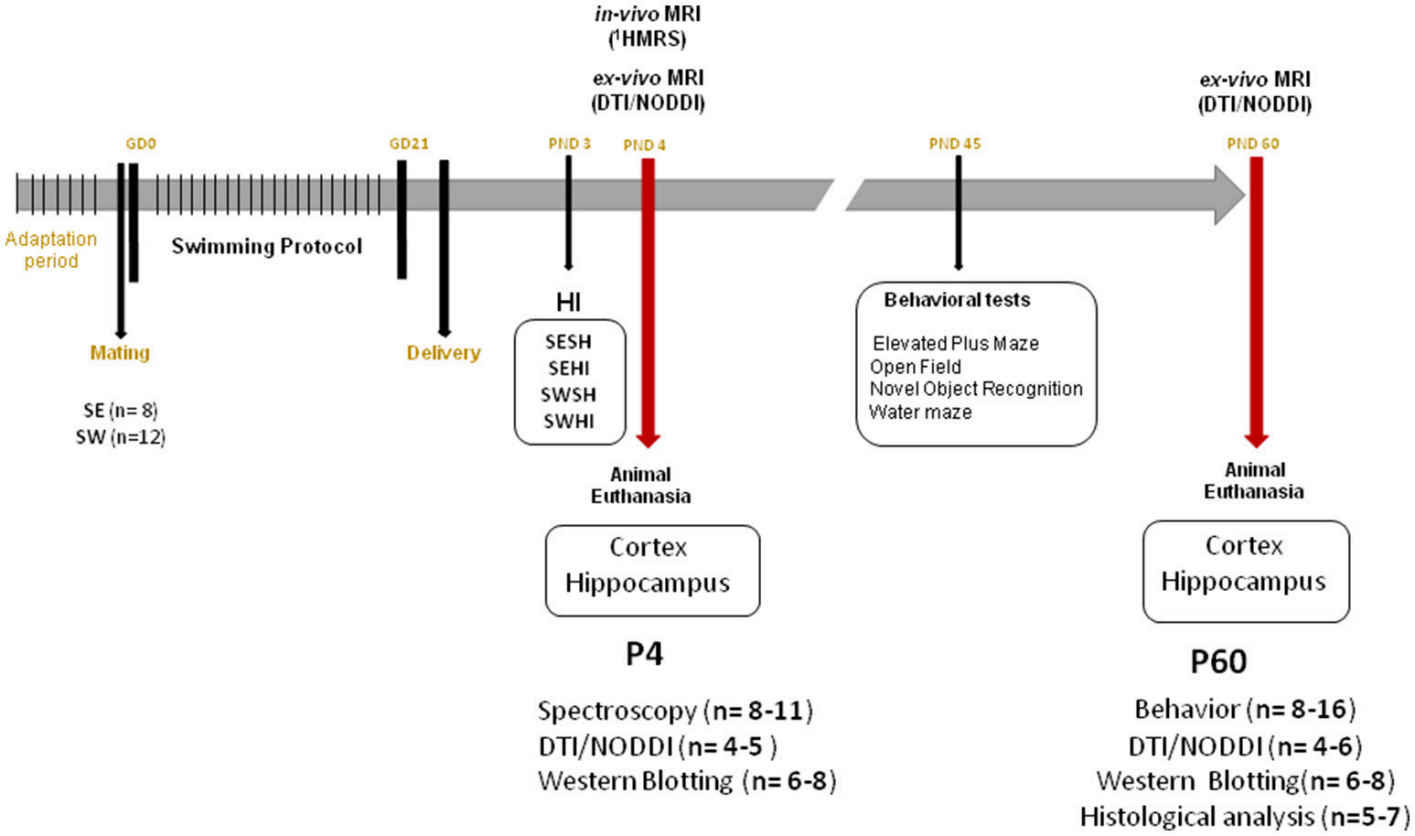
Experimental timeline. GD, Gestational Day; HI, Hypoxia-Ischemia; PND, Postnatal day; DTI, Diffusion Tensor Imaging; NODDI, Neurite Orientation Dispersion and Density Index; SE, Sedentary; SW, Swimming.

The Geneva State Animal Ethics Committee and the Swiss Federal Veterinary Service approved this study under GE/132/15 license. Male and female Wistar rats were ordered from Charles River Laboratories (L'Arbresle, France). Animals were housed under standard laboratory conditions (12-h-light, 12-h-dark cycle and room temperature at 22 ± 1°C). One week prior to mating, the females were distributed to Sedentary (SE) or Swimming (SW) group and acclimated to a black circular acrylic water tank (200 cm diameter) filled with warm water at 32 ± 1°C (25 cm depth for SW group). A 100 cm diameter tank (made with the same black plastic material) and kept empty was used for the exposition of the SE group. Standard rat chow and water was provided *ad libitum*. Sedentary (SE) and Swimming (SW) females were mated (Gestational day 0—GD0) and kept two per cage until GD20. The timeline of the experiments is shown in Figure 1.

### Swimming protocol

The swimming protocol consisted in a training period of 4 days swimming with increasing time exposures in the tank (5, 10, 15, and 20 min/day) previously to mating. Then it consisted of 20 min daily sessions from 1st to the 21st day of pregnancy (50). After each session, the animals were dried with a face towel and kept under an infrared lamp until completely dried. From GD 20 until delivery, they were housed individually in a clean standard cage. The control non-swimming animals were daily exposed to an empty circular open field (measures 100 cm diameter and 45 width) to be manipulated by the experimenters and exposed to a different environment (same material as the swimming tank). SE (*n* = 8) and SW (*n* = 12).

### Neonatal hypoxia-ischemia

At PND1 the newborn animals were counted and the litters were culled to between 8 and 12 pups to avoid differences in animal weights. Both sexes were used for the procedure in a rate of 50% each. At PND3 pups were submitted to mild to moderate hypoxic-ischemic injury as previously described (16, 21, 23). Briefly, under isoflurane anesthesia (4% induction and 1.5–2.0% maintenance), the right carotid artery was isolated from the vagus nerve and surrounding tissue and permanently occluded with 6.0 silk thread. The surgical access was closed with Histoacryl™ and Steri-strip™. After a 30 min recovery period in a chamber at 37°C with room air, the flux of room air was replaced by a 2 l/min of 6% O_2_ at 37°C during 30 min to induce hypoxia. Sham animals were anesthetized, had the incision without carotid occlusion or hypoxia. For all experiments, SE and SW litters were processed in parallel. In total, 4 groups were assessed: (1) Sedentary-Sham (SESH), (2) Sedentary-Hypoxic-Ischemic (SEHI), (3) Swimming-Sham (SWSH) and (4) Swimming-Hypoxic-Ischemic (SWHI).

### Magnetic resonance

MR experiments were performed on an actively-shielded 9.4T/31 cm magnet (Agilent/Varian/Magnex) equipped with 12 cm gradient coils (400 mT/m, 120 μs) with a quadrature transceive 20 mm surface RF coil as previously described (16, 51).

### [^1^-H] MR spectroscopy

For ^1^H-MRS (24 h after injury), the rats were continuously anesthetized under a flow of 1.5–2% isoflurane in O_2_. Body temperature was kept at 37 ± 0.5°C during the entire procedure. For a better characterization of the injury as well as to identify more precisely the effects of maternal swimming, animals were categorized according to the lesion severity using the presence of a hypersignal in the cortex on T_2_W images. At PND4, 24 h after injury, after automatic FASTMAP shimming, spectra acquisition on a voxel of interest of 1.5 × 1.5 × 2.5 mm^3^ within the parietal cortex was performed for the 4 groups using an ultra-short echo time (TE/TR = 2.7/4,000 ms) SPECIAL spectroscopy method (52). Proton spectra were analyzed with LCModel (53) providing the neurochemical profile of the right injured hemisphere and in the right hemisphere for the controls, for SE and SW groups. The results provided the quantification of the following metabolite concentrations: aspartate (Asp), alanine (Ala), ascorbate (Asc), creatine (Cr), phosphorylcholine (PCho), phosphocreatine (PCr), γ-aminobutyric acid (GABA), glutamate (Glu), glutamine (Gln), glutathione (GSH), glycine (Gly), lactate (Lac), macromolecules (Mac), myoinositol (Ins), N-acetylaspartate (NAA), N-acetylaspartylglutamate (NAAG), phosphoethanolamine (PE) and taurine (Tau).

### Diffusion tensor imaging (DTI)/neurite orientation dispersion index (NODDI)

At PND4 and PND60 (*n* = 4–6 animals/group per time point), rats were sacrificed and brains were paraformaldehyde-fixed for subsequent *ex vivo* MRI with a 2.5 mm diameter birdcage coil. A multi-b-value shell protocol was acquired using a spin-echo sequence (FOV = 21 × 16 mm^2^, matrix size = 128 × 92, 12 slices of 0.6 mm, 3 averages with TE/TR = 45/2,000 ms). 96 DWI were acquired, 15 b_0_ images and 81 separated in 3 shells (non-collinear and uniformly distributed in each shell) with number of directions/b-value in s/mm^2^: 21/1750, 30/3,400 and 30/5,100, respectively. Acquired data were fitted using the NODDI toolbox (54). At PND4 and PND60, three different brain regions were identified: cortex (Cx), corpus callosum (CC) and external capsule (EC). DTI derived parameters [Axial diffusivity (AD), Radial diffusivity (RD), Mean diffusivity (AD) and Fractional anisotropy (AD)] as well as NODDI derived parameters (intra-neurite volume faction (*f*_*icvf*_), isotropic volume fraction (*f*_*iso*_) and orientation dispersion index (ODI) were averaged in the different regions assessed.

### Behavioral analysis

Given that HI lesion involve several regions, including sensorimotor cortex, and hippocampus, as from 45 days of age animals were tested in the Elevated Plus Maze (EPM), Open Field (OF), Novel Object Recognition (NOR) and Morris Water Maze (MWM). All animals performed the tasks in the above cited order. The apparatuses were thoroughly cleaned between every animal and the male rats were tested first. All behavioral procedures were performed between 9 a.m. and 4 p.m. The same investigators performed all experimental sessions in a controlled light, temperature and sound room. After each trial, the apparatuses were cleaned with a 70% ethanol solution (24).

#### Elevated plus maze (EPM)

The elevated plus maze, allowing to measure anxiety, is a device with two open arms (50 × 10 cm), surrounded by an edge of 0.5 cm and two closed arms (50 × 10 × 15 cm) and the central area measuring 10 cm^2^. The maze was elevated to a height of 70 cm. Each rat was placed at the center of the apparatus facing one enclosed arm. The test was video recorded for 5 min and using the ANY-Maze software (Stoelting Co., USA) the number of entries into open or closed arms and the total time spent in each arm was recorded. An entry was defined by placing the four paws into an arm (24).

#### Open field (OF)

The test allows the observation of exploratory activity of animals in a novel environment. The apparatus consists of a circular wooden chamber (100 cm diameter × 30 cm high wall) with a floor divided into 21 fields. Using ANY-Maze software, the open field test was video recorded during 5 min. The latency to leave the central circle, number of crossings and rearings were considered as indicative of spontaneous motor activity.

#### Novel object recognition (NOR)

The novel object recognition task assesses declarative memory (55). In the first phase of the test, each animal was confronted with two different objects, placed in an open-field box (the same used for the open field test) and the time of object exploration was registered for 5 min. Following this phase, the rodent was removed from the open-field box and put in another separate box for a period of 5 min. In the second phase, each animal was exposed to two objects placed in the same open-field box: one familiar object, used in the first phase, and one novel object. The time spent exploring the novel object and the familiar object was measured. A discrimination index was calculated in the test session (second), as follows: the difference in exploration time divided by the total time spent exploring the two objects (B – A/B + A, where B is the new object and A is the familiar object) (56).

#### Morris water maze (MWM)

Spatial memory was tested in the Morris water maze task as previously described (24). Rats entered the pool facing the wall and from a start position designated as N, S, W or E. All rats accomplished four trials/day, on 5 consecutive days, with a 10-min inter-trial interval and every starting point was used in a different order each day. The latency to find the platform during each trial was measured as a learning index. During the five training days, the platform remained at the same location. A probe test (without the platform) was performed on the 6th day and parameters such as latency to cross the platform zone, time spent in platform quadrant, time spent in the opposite platform quadrant and total distance traveled were assessed using the ANY-Maze software.

### Protein analysis

For the western blotting analysis, pups were sacrificed at either PND4 or PND60 and brain structures were quickly collected on ice and the right cortex and hippocampus (ipsilateral to the lesion) were dissected out and frozen in RIPA buffer (Cell Signaling, 9806S) at −20°C. Structures were sonicated and the protein concentration was determined using a Bradford assay. Proteins (25 μg) were separated by SDS-PAGE, transferred on nitrocellulose membrane and analyzed by immunoblotting. The primary antibodies were diluted (1:1,000) in blocking solution containing 0.1% casein (Sigma-Aldrich, C8654). PND4 brains were analyzed for neurons (NeuN and DCX), astrocytes (GFAP), (GLT-1) and glutamine synthetase, oligodendrocytes progenitors (NG2), microglia/macrophages (CD11b and Iba-1), apoptosis (fractin and cleaved caspase 3), neurotrophic factors (VEGF and BDNF) and the BDNF receptor Tyrosin Kinase (Trk-B). For the PND60 assessment, the membranes were incubated with the primary antibodies: NeuN, GFAP, MBP, BDNF, VEGF, and Trk-B. After overnight incubation with the primary antibody, the following secondary antibodies (1:10,000) were applied: goat anti-mouse IgG conjugated with IRDye 680 (LI-COR, B70920-02), goat anti-rabbit IgG conjugated with IRDye 800 (LI-COR, 926- 32210) and donkey anti-guinea pig IgG conjugated with IRDye 800 (LI-COR, 926-32411). Protein bands were visualized using the Odyssey Infrared Imaging System (LI-COR). ImageStudio™ Lite (LI-COR) was used to measure the optical densities of the protein signals on scans. The relative optical density was calculated using the optical density of protein signals divided by the optical density of a loading control (actin or βIII-tubulin) and expressed as a percentage of values obtained compared to the SESH group (100%) (*n* = 6–8 animals/group). The list of the antibodies used is in Table 1.

**Table 1:** List of the antibodies used.

**Antibody**	**Company**	**Reference**	**Host**	**MW**
Actin	Millipore	MAB1501	Mouse	42 kDa
BDNF	Abcam	ab46176	Rabbit	28 kDa
CD11b	Serotec	MCA275G	Mouse	160 kDa
ccaspase 3	Cell signal	9661	Rabbit	19 kDa
DCX	Abcam	ab18723	Rabbit	45 kDa
Fractin	Millipore	AB3150	Rabbit	32 kDa
GFAP	Sigma	G6171	Mouse	55 kDa
GFAP	Dako	Z0334	Rabbit	55 kDa
GLT-1	Abcam	ab106289	Rabbit	62 kDa
Glut synth	Abcam	73593	Rabbit	42 kDa
Iba-1	Abcam	ab5076	Goat	17 kDa
NeuN	Milllipore	MAB377	Mouse	46/48 kDa
NG2	Milllipore	MAB5384	Mouse	262 kDa
Trk-B	Abcam	ab18987	Rabbit	92 kDa
Tubulin	Abcam	ab18207	Rabbit	50/55 kDa
VEGF	Abcam	ab1316	Mouse	24/45 kDa

### Statistical analysis

All statistical analysis was performed using SPSS 19.0 for Windows (SPSS Inc., Chicago, IL, USA). Data are presented as mean ± standard error of the mean (SEM). Non-parametric data was analyzed by Kruskall-Wallis followed by Mann-Whitney test for multiple comparisons. One-way ANOVA followed by Duncan's *post-hoc* was used to compare differences among the groups presenting normal distribution. The significance was accepted when *p* < 0.05.

## Results

Pregnant rats were weighed daily from GD1 to GD21 before swimming sessions or exposition to the open field, in the SE group. Animals in both swimming and sedentary groups gained weight during pregnancy [*F*_(4, 72)_ = 158.96; *p* < 0.05] with no significant differences between the groups [*F*_(4, 72)_ = 0.329; *p* = 0.858] (data not shown). On delivery, the swimming group had an average of 10 pups whereas the control group litters averaged 11 (no statistical difference was observed—data not shown). The litters were sorted to have 50% rats of each sex distributed equally among the four groups. Pups weight was modulated by maternal swimming at PND14 [*F*_(3, 72)_ = 4.74, *p* < 0.05], PND21 [*F*_(3, 72)_ = 6.38, *p* < 0.05], PND45 [*F*_(3, 72)_ = 3.06, *p* < 0.05] and PND60 [*F*_(3, 72)_ = 4.04, *p* < 0.05], in which animals from the swimming groups (SW) had increased body weights compared to the sedentary (SE) ones. There was no effect of HI *per se* on this measure (data not shown).

### ^1^HMR spectroscopy

FASTMAP shimming (first-order and second-order correction of the magnetic field homogeneity) enabled to obtain a very good-quality of spectra in a volume of 12 μl in the parietal cortex. The average signal-to-noise ratio calculated on all acquired spectra was 13.9 ± 1.9. Table 2 shows the concentration of the 18 metabolites assessed using the spectral analysis and absolute quantification by LCModel (53). Significant differences were observed in the concentration of Gln and the ratio Gln/Glu between SESH and SWSH groups, as well as trends to a decrease in NAAG (*p* = 0.07), PE (*p* = 0.06), Mac (*p* = 0.06), Glu+Gln (*p* = 0.07) in the SWSH group, evidencing the effect of maternal swimming on pup's brain metabolism. 24 h after HI, concentration of almost all the quantified metabolites decreased in the cortical tissue of the SEHI group compared to the SESH group including [PCho], [Cr], [PCr], [Glu], [GSH], [Ins], [NAA], [NAAG], [Tau], [Asc], [PE], [Mac], [Glu+Gln], [GPC+PCho], and [Cr+PCr]. Maternal swimming prevented the decrease of [PCho], [PCr], [Ins], [NAAG], [NAA+NAAG], and [Lac/NAA] in the SWHI group compared to the SWSH, evidencing preservation of the energetic metabolism induced by maternal swimming. Interestingly, no increase in [Lac] (marker of anaerobic metabolism) was observed in the injured groups pointing to a milder injury level compared to previous studies performed by the group (16, 57).

**Table 2:** Concentrations ± SEM for the metabolites in the [1H] MRS.

	**SESH (*n* = 8)**	**SWSH (*n* = 8)**		**SEHI (*n* = 11)**		**SWHI (*n* = 11)**
Ala	0.67 ± 0.27	0.47 ± 0.17	–	0.42 ± 0.24	–	0.48 ± 0.32	–
+Asp	1.14 ± 0.43	1.04 ± 0.33	–	0.94 ± 0.18	–	1.06 ± 0.32	–
PCho	1.66 ± 0.18	1.53 ± 0.19	–	1.27 ± 0.28^*^	↓	1.40 ± 0.26	–
Cr	2.64 ± 0.40	2.41 ± 0.32	–	1.91 ± 0.48^*^	↓	1.76 ± 0.34^*^	↓
PCr	3.09 ± 0.43	3.17 ± 0.34	–	2.58 ± 0.48^*^	↓	2.78 ± 0.62	–
GABA	0.85 ± 0.22	0.88 ± 0.26	–	0.67 ± 0.32	–	0.67 ± 0.25	–
Gln	2.01 ± 0.36	1.57 ± 0.43^#^	↓	1.95 ± 0.37	–	1.70 ± 0.27	–
Glu	4.42 ± 0.81	4.81 ± 0.44	–	3.65 ± 0.71^*^	↓	3.73 ± 0.87^*^	↓
GSH	1.17 ± 0.25	1.14 ± 0.13	–	0.88 ± 0.22^*^	↓	0.84 ± 0.33^*^	↓
Gly	1.91 ± 0.57	1.78 ± 0.40	–	1.48 ± 0.37	–	1.70 ± 0.47	–
Ins	1.69 ± 0.27	1.50 ± 0.32	–	0.98 ± 0.52^*^	↓	1.02 ± 0.55	–
Lac	1.86 ± 0.57	1.56 ± 0.34	–	2.33 ± 1.14	–	1.83 ± 0.41	–
NAA	1.82 ± 0.26	1.83 ± 0.27	–	1.55 ± 0.20^*^	↓	1.56 ± 0.34^*^	↓
Tau	17.97 ± 1.44	17.09 ± 1.32	–	14.62 ± 1.66^*^	↓	14.58 ± 1.7^*^	↓
Asc	5.15 ± 0.56	4.90 ± 0.39	–	3.42 ± 0.64^*^	↓	3.74 ± 0.56^*^	↓
NAAG	1.15 ± 0.20	0.99 ± 0.13	↓ 0.07	0.94 ± 0.15^*^	↓	1.00 ± 0.19	–
PE	5.27 ± 0.41	4.95 ± 0.23	↓ 0.06	4.30 ± 0.46^*^	↓	4.28 ± 0.73^*^	↓
Mac	1.70 ± 0.12	1.61 ± 0.06	↓ 0.06	1.42 ± 0.13^*^	↓	1.42 ± 0.15^*^	↓
NAA+NAAG	2.97 ± 0.35	2.82 ± 0.36	–	2.49 ± 0.27^*^	↓	2.57 ± 0.36	–
Glu+Gln	6.44 ± 1.05	6.39 ± 0.72	↓ 0.07	5.60 ± 0.77	–	5.44 ± 0.96^*^	↓
GPC+PCho	2.02 ± 0.22	1.84 ± 0.14	–	1.51 ± 0.35^*^	↓	1.59 ± 0.28^*^	↓
Cr+PCr	5.74 ± 0.55	5.58 ± 0.37	–	4.49 ± 0.51^*^	↓	4.54 ± 0.63^*^	↓
Glu/Gln	2.23 ± 0.36	3.24 ± 0.82^#^	↑	1.95 ± 0.58^?^	–	2.23 ± 0.55^*^	↓
PCr/Cr	1.20 ± 0.28	1.35 ± 0.29	–	1.49 ± 0.67	–	1.65 ± 0.54	–
Lac/NAA	1.03 ± 0.30	0.87 ± 0.25	–	1.52 ± 0.77^*^	↑	1.29 ± 0.49	–

*Differences among the different groups (SESH, SEHI, SWSH and SWHI) 24 h post-HI (p < 0.05*,

* *Effect of injury - HI vs. SH*,

# *SESH vs. SWSH. Ala, Alanine*;

*+Asp, Aspartate; PCho, phosphocholine; Cr, creatine; PCr, phosphocreatine; GABA, gama aminobutyric acid; Gln, glutamine; Glu, glutamate; GSH, glutathione; Gly, glycine; Ins, Myoinositol; Lac, lactate; NAA, N-acetylaspartate; Tau, taurine; Asc, Ascorbate; NAAG, N-acetylaspartylglutamate; PE, phosphoethanolamine; Mac, macromolecules*.

### Western blotting

#### Expression of cell markers

##### Neurons

**Figure 2:**
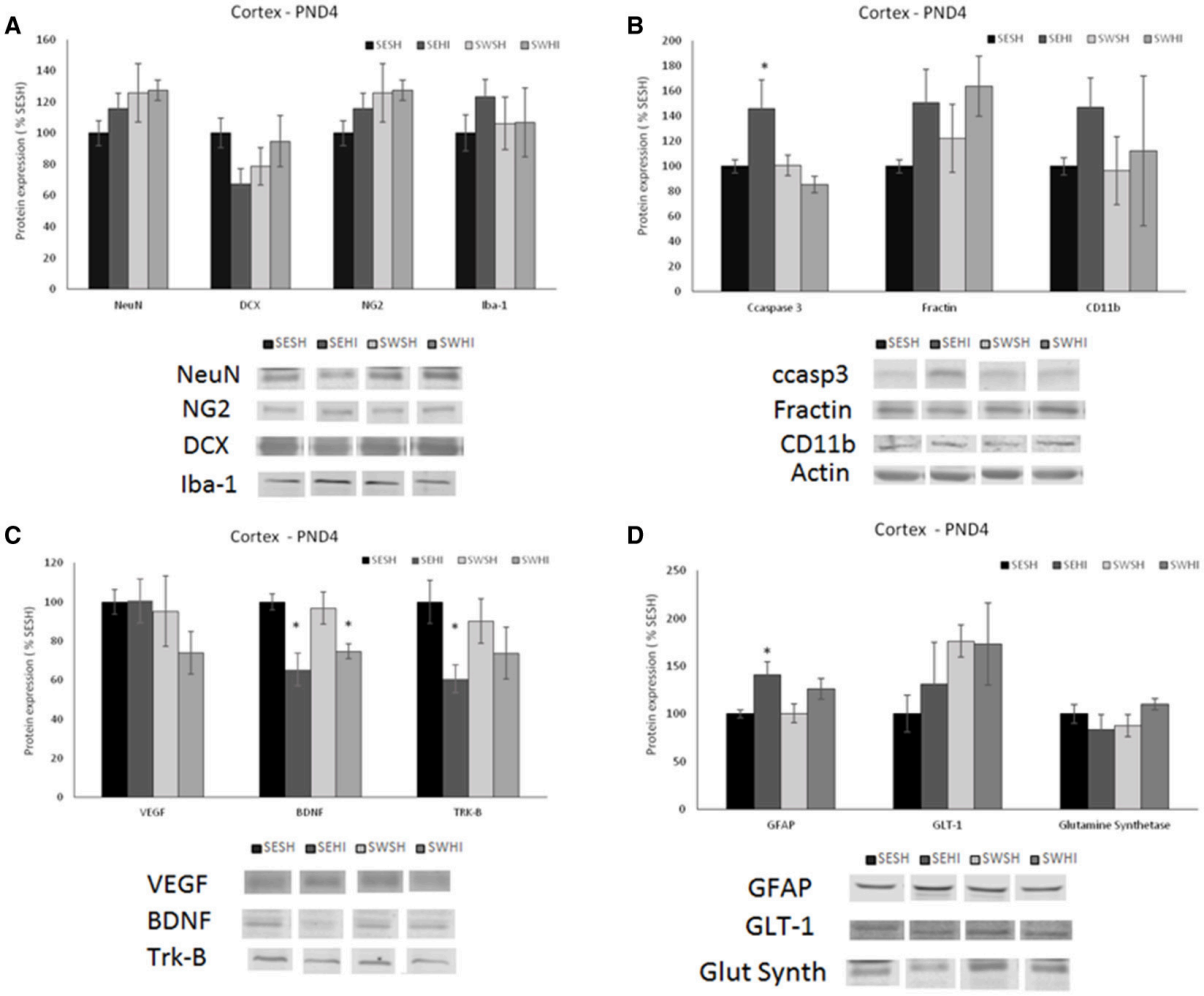
Effect of pregnancy swimming on the pup's protein levels in right cortex extracts 24 h following HI (PND4). Bars graph represent immunoblots of **(A)** neurons (NeuN) and migrating neurons Doublecortin (DCX), oligodendrocytes progenitor (NG2) and microglia (Iba-1); **(B)** cell death (ccaspase 3 and fractin) and inflammation (CD11b), **(C)** neurotrophins VEGF and BDNF and the TRK-B receptor and **(D)** astrogliosis (GFAP), glutamate receptor GLT-1 and glutamine synthetase enzyme in the four experimental groups: sedentary sham (SESH), sedentary hypoxic-ischemic (SEHI), swimming sham (SWSH) and swimming hypoxic-ischemic (SWHI). WB results are plotted normalized to SESH level expression (100%) (mean ± SEM). Significance testing was determined using one-way ANOVA followed by Duncan's *post hoc* and was performed on Actin or βIII-tubulin normalized data. *HI vs. its respective SH group, Significance accepted when *p* < 0.05.

**Figure 3:**
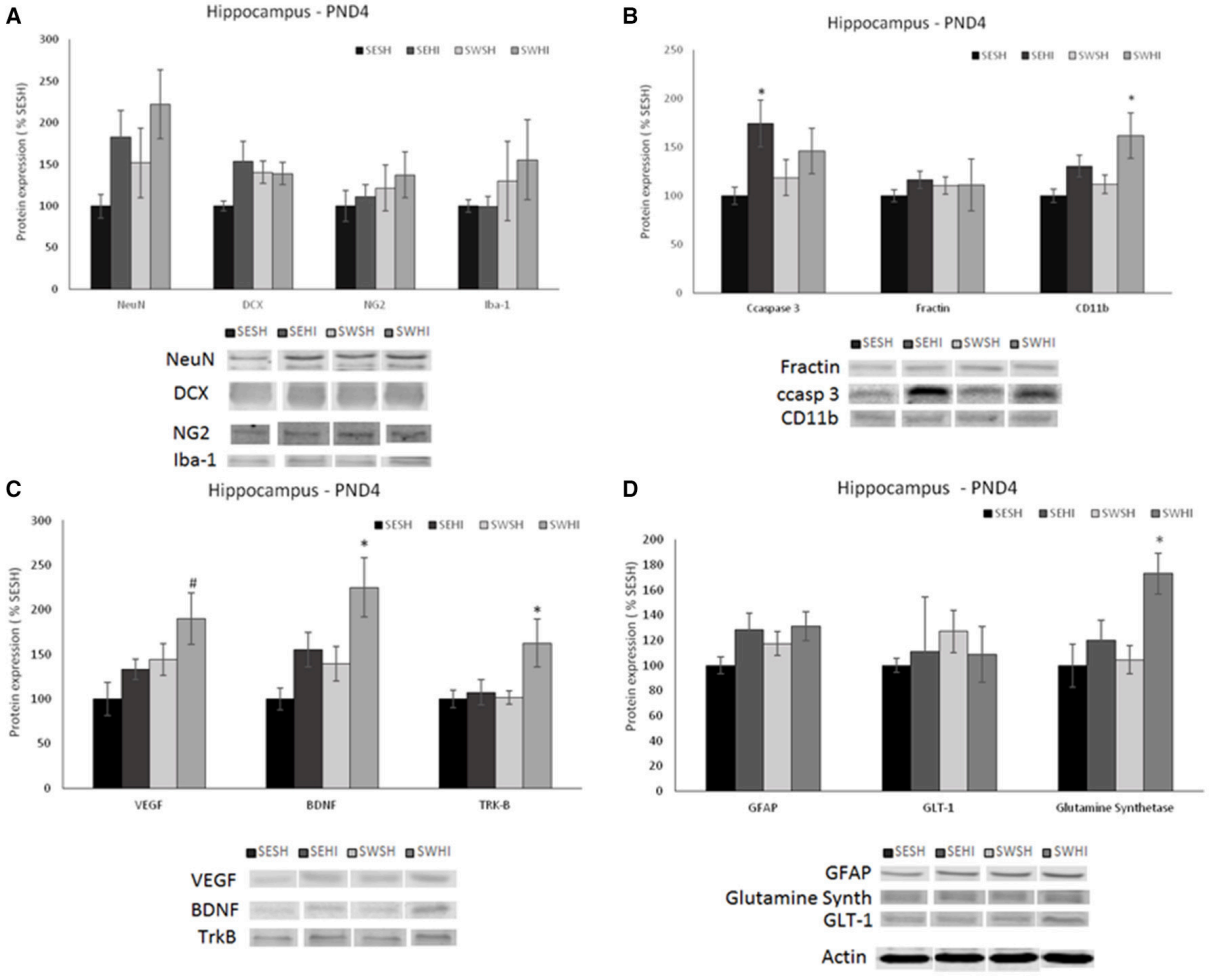
Effect of pregnancy swimming on the pup's protein levels on the right hippocampus extracts 24 h following HI (PND4). Bars graph represent immunoblots of **(A)** neurons (NeuN) and migrating neurons Doublecortin (DCX), oligodendrocytes progenitors (NG2) and microglia (Iba-1); **(B)** cell death (ccaspase 3 and fractin) and inflammation (CD11b), **(C)** neurotrophins VEGF and BDNF and the TRK-B receptor and **(D)** astrogliosis (GFAP), glutamate receptor GLT-1 and glutamine synthetase enzyme in the four experimental groups: sedentary sham (SESH), sedentary hypoxic-ischemic (SEHI), swimming sham (SWSH) and swimming hypoxic-ischemic (SWHI). WB results are plotted normalized to SESH level expression (100%) (mean ± SEM). Significance testing was determined using one-way ANOVA followed by Duncan's *post-hoc* and was performed on Actin or βIII-tubulin normalized data. *HI vs. its respective SH group. ^#^SWHI vs. SESH and SEHI. Significance accepted when *p* < 0.05.

No differences were observed in the expression of mature (NeuN) [*F*_(3, 27)_ = 1.012, *p* = 0.405] nor migrating (DCX) neurons in the cortex [*F*_(3, 27)_ = 1.950, *p* = 0.148] (Figure 2A) nor in the hippocampus [*F*_(3, 27)_ = 2.428, *p* = 0.900; *F*_(3, 27)_ = 1.715, *p* = 0.191] (Figure 3A).

##### Oligodendrocytes progenitors (NG2)

No difference was observed in NG2 expression neither in the cortex [*F*_(3, 27)_ = 0.936, *P* = 0.43] nor in the hippocampus [*F*_(3, 26)_ = 0.55, *P* = 0.65; Figures 2A, 3A].

##### Microglia (Iba-1)

No differences were observed in the protein expression of microglial cells (Iba-1) neither in the cortex [*F*_(3, 27)_ = 0.419, *P* = 0.741] nor in the hippocampus [*F*_(3, 27)_ = 0.638, *P* = 0.59; Figures 2A, 3A].

##### Astrocytes

GFAP was increased in the cortex of the SEHI compared to SESH group [*F*_(3, 28)_ = 3.52, *P* = 0.02] evidencing the early astrogliosis caused by HI and the protection offered by the maternal swimming (Figure 2D). No alteration was observed in the right hippocampus [*F*_(3, 27)_ = 1.442, *p* = 0.255; Figures 3D]. Glutamate transporter 1 (GLT-1) was not altered in the cortex [*F*_(3, 25)_ = 0.871, *p* = 0.471] nor in the hippocampus [*F*_(3, 26)_ = 0.152, *p* = 0.927; Figures 2D, 3D]. The enzyme glutamine synthetase was not altered in the cortex [*F*_(3, 26)_ = 1.393, *p* = 0.270; Figure 2D]. In the hippocampus (Figure 3D) the enzyme expression was increased in the SWHI compared to the other groups [*F*_(3, 26)_ = 4.65, *p* = 0.01].

#### Apoptosis and inflammation

##### Cleaved caspase 3 (ccasp3)

As shown in Figures 2B, 3B, in the cortex, the expression of cleaved caspase-3 was increased in the SEHI compared to all other groups [*F*_(3, 29)_ = 3.63, *p* = 0.02] ipsilateral to injury at 24 h. In the hippocampus [*F*_(3, 26)_ = 2.89, *P* = 0.05], ccasp3 was increased in the SEHI compared to the SESH group. Maternal swimming prevented the apoptotic cell death increase in the SWHI group in both structures.

##### Fractin

No differences were observed neither in the cortex [*F*_(3, 27)_ = 1.348, *p* = 0.282] nor in the hippocampus [*F*_(3, 27)_ = 0.618, *p* = 0.610; Figures 2B, 3B].

##### Macrophages/monocytes (CD11b)

Despite the increase observed in CD11b expression in the SEHI group in the cortex of the group, no significant differences were observed [*F*_(3, 25)_ = 0.525, *p* = 0.669; Figure 2B]. In the hippocampus, there was a significant increase in the protein expression in the SWHI group compared to SESH and SWSH groups [*F*_(3, 27)_ = 3.243, *p* = 0.04] evidencing an early inflammatory reaction (Figure 3B).

### Expression of neurotrophins (VEGF and BDNF and the receptor tyrosin kinase receptor-B (Trk-B)

Figures 2C, 3C show neurotrophins expression in cortex and hippocampus at PND4. No significant differences observed in VEGF expression in the cortex (Figure 2C). Figure 2C shows a significant decrease in BDNF expression in the cortex of HI groups (sedentary and swimming) [*F*_(3, 25)_ = 6.37, *P* = 0.003]. However, Tyrosin kinase B (Trk-B) receptor expression was decreased only in this SEHI group in the structure [*F*_(3, 24)_ = 3.20, *p* = 0.04] evidencing an effect due to swimming.

In the hippocampus, SWHI groups had an increase in VEGF expression compared to SESH and SEHI groups [*F*_(3, 26)_ = 3.28, *p* = 0.03; Figure 3D]. BDNF expression was increased in the SWHI compared to SESH and SWSH groups [*F*_(3, 27)_ = 4.94, *P* = 0.008; Figure 3C] and TRK-β protein expression [*F*_(3, 27)_ = 3.23, *p* = 0.04] in the SWHI group (Figure 3C) compared to all other groups.

At PND60, no differences were observed in the expression of neurons (NeuN) in the cortex [*F*_(3, 27)_ = 1.315, *p* = 0.293] and hippocampus [*F*_(3, 26)_ = 1.915, *p* = 0.155], astrocytes (GFAP) in the cortex [*F*_(3, 26)_ = 0.377, *p* = 0.770] and hippocampus [*F*_(3, 27)_ = 0.829, *p* = 0.491], myelin (MBP) in the cortex [*F*_(3, 24)_ = 0.215, *p* = 0.885] and hippocampus [*F*_(3, 27)_ = 0.350, *p* = 0.789], BDNF in the cortex [*F*_(3, 26)_ = 0.208, *p* = 0.890] and hippocampus [*F*_(3, 26)_ = 0.409, *p* = 0.748] and Trk-B in the cortex [*F*_(3, 27)_ = 0.843, *p* = 0.484] and hippocampus [*F*_(3, 26)_ = 0.699, *p* = 0.562; Figure 7, upper panels]. The expression of VEGF was significantly increased in the hippocampus of the SEHI group compared to the other groups [*F*_(3, 25)_ = 3.48, *P* = 0.03; Figure 7, upper panels]. No differences in VEGF in the cortex were observed [*F*_(3, 26)_ = 0.620, *p* = 0.620].

### Behavioral testing

Table 3 shows the Elevated Plus Maze (EPM) and Open Field (OF) analysis. In the EPM, rats from the SEHI group had a trend to spend more time in the closed arms (*p* = 0.06) than in the open arms indicative of anxiety (16 s compared to 8.7 s), however, the latencies were not statistically significant. Together, these results suggest an anxiogenic profile in SEHI rats prevented by swimming. No increased locomotor or exploratory activity were observed in the OF in the number of neither crossings (horizontal) nor rearings (vertical) exploration that could indicate hyperactivity induced by HI. The cognitive capabilities were tested using non-spatial and spatial tests. The non-spatial testing consisted of the NOR test, based on the inherited exploratory behavior of novelty in rodents. We did not detect impairment in the non-spatial memory, as sham and HI animals explored equally both objects. When spatial memory was evaluated in the MWM, repeated measures ANOVA indicated a significant effect of groups [*F*_(1, 67)_ = 7.66, *p* < 0.05] and in the days of training [*F*_(1, 67)_ = 7.66, *p* < 0.05]; also, SWHI showed decreased escape latencies to find the platform on the 5th day of training [*F*_(3, 44)_ = 3.10, *p* < 0.05] as well as on the latency to reach the platform location in the Probe trial (test day) [*F*_(3, 44)_ = 3.01, *p* < 0.05] corresponding to learning impairments in the SEHI group, not observed in the SWHI group (Figure 5).

**Table 3:** Behavioral analysis at adult age.

	**SESH**	**SEHI**	**SWSH**	**SWHI**
**ELEVATED PLUS MAZE**				
First open arm entry (s)	50.6 ± 22.7	37.6 ± 10.0	46.4 ± 16.0	50.6 ± 38.6
Open arm (s)	8.8 ± 3.6	16.7 ± 7.1	11.1 ± 4.4	12.1 ± 5.8
Closed arm (s)	267.8 ± 7.4	234.9 ± 9.0	262.2 ± 7.6	260.0 ± 9.1
Ratio open/closed	0.1 ± 0.03	0.3 ± 0.05	0.2 ± 0.03	0.2 ± 0.04
Risk evaluation	7.5 ± 0.8	11.1 ± 1.0	8.3 ± 0.7	8.1 ± 0.9
**OPEN FIELD**				
Crossings	172.5 ± 8.5	196.7 ± 8.8	172.3 ± 6.6	176.9 ± 16.3
Rearings	15.7 ± 2.3	13.9 ± 2.6	13.9 ± 1.8	19.8 ± 4.5
Latency	1.6 ± 0.5	2.2 ± 1.1	1.3 ± 0.4	1.1 ± 0.4
**NOVEL OBJECT RECOGNITION**				
Index Phase I	0.15 ± 0.06	0.007 ± 0.02	0.12 ± 0.04	0.230 ± 0.098
Index Phase II	0.33 ± 0.08	0.36 ± 0.05	0.36 ± 0.06	0.373 ± 0.12

*Data are expressed as mean ± SEM (n = 8–16). The results were analyzed by one-way ANOVA followed by Duncan's post-hoc test. Significance was accepted when p < 0.05. No differences were observed*.

### Microstructure evaluation—DTI/NODDI

**Figure 4:**
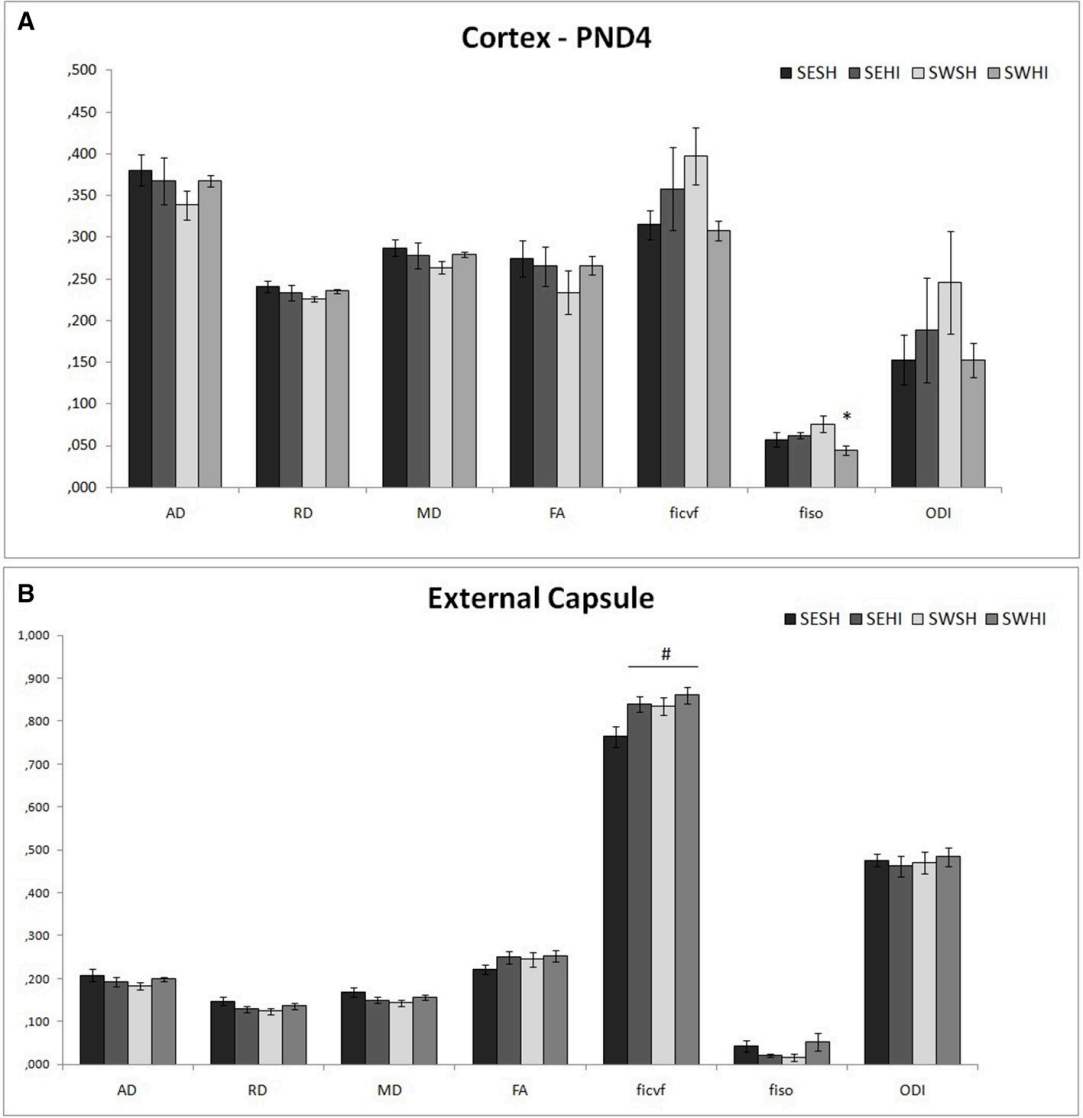
DT-MRI microstructural white matter alterations at PND4. Histograms of mean values ± SEM of the DTI derived parameters at PND4 in the cortex **(A)**, and external capsule **(B)**. Axial Diffusivity (AD), Radial Diffusivity (RD), Median diffusivity (MD), fractional anisotropy (FA) and NODDI estimates: intraneurite volume fraction (f_*icvf*_), cerebrospinal volume fraction (*f*_*iso*_) and orientation dispersion index (ODI) for SESH, SEHI, SWSH and SWHI groups. *SWHI vs. SWSH. ^#^SESH vs. all other groups.

Direction encoded brain color maps of the rat pups are presented in **Figure 6**. The excellent SNR and resolution quality (70 μm in-plane) of these images allowed an accurate estimation of diffusion tensor derived parameters. No obvious visual differences were observed between the maps (i.e., thinner cortex in the injured hemisphere due to cortical loss following injury) at the intervals studied. At PND4, FA measurements are presented as mean values of two different brain regions (Cortex and External capsule) (Figures 4A,B). In the cortex *f*_*iso*_ [*F*_(3, 17)_ = 3.44, *p* = 0.04] was reduced in the SWHI group compared to the SWSH. In the external capsule, *f*_*icvf*_ [*F*_(3, 17)_ = 4.01, *p* = 0.03] was reduced in the SESH group compared to the other groups. No differences were observed in the FA or in the ODI in any of the structures at PND4.

**Figure 5:**
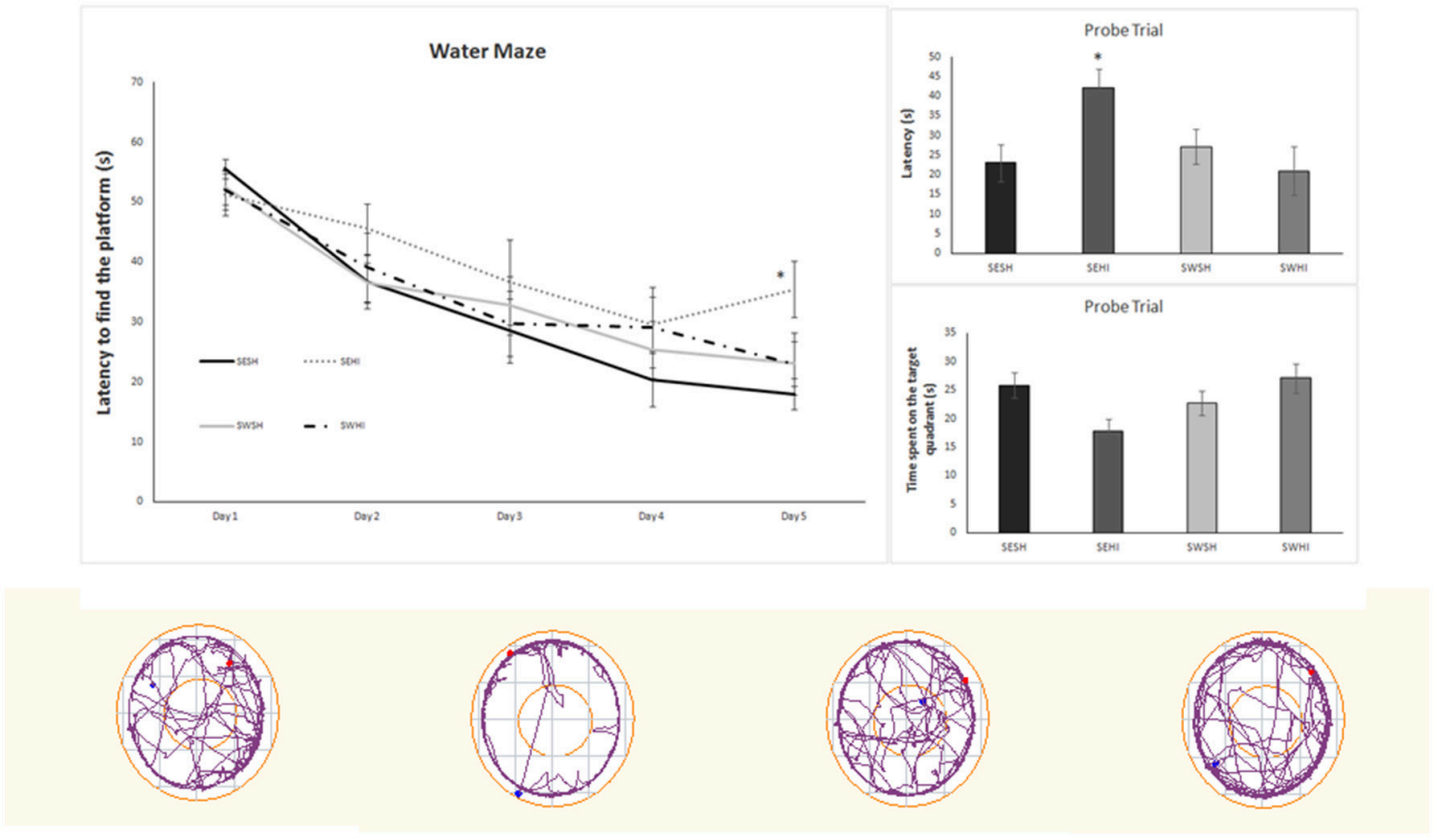
Water Maze performance during the 5 days of training (left upper panel). (Right upper panels) - performance on the probe trial. Data are expressed as mean ± SEM (*n* = 8–16). Lower panels show the representative plots of the Probe Trial. The results were analyzed by two-way ANOVA followed by Duncan's *post-hoc* test. Significance was accepted when *p* < 0.05. *SEHI vs. SESH.

**Figure 6:**
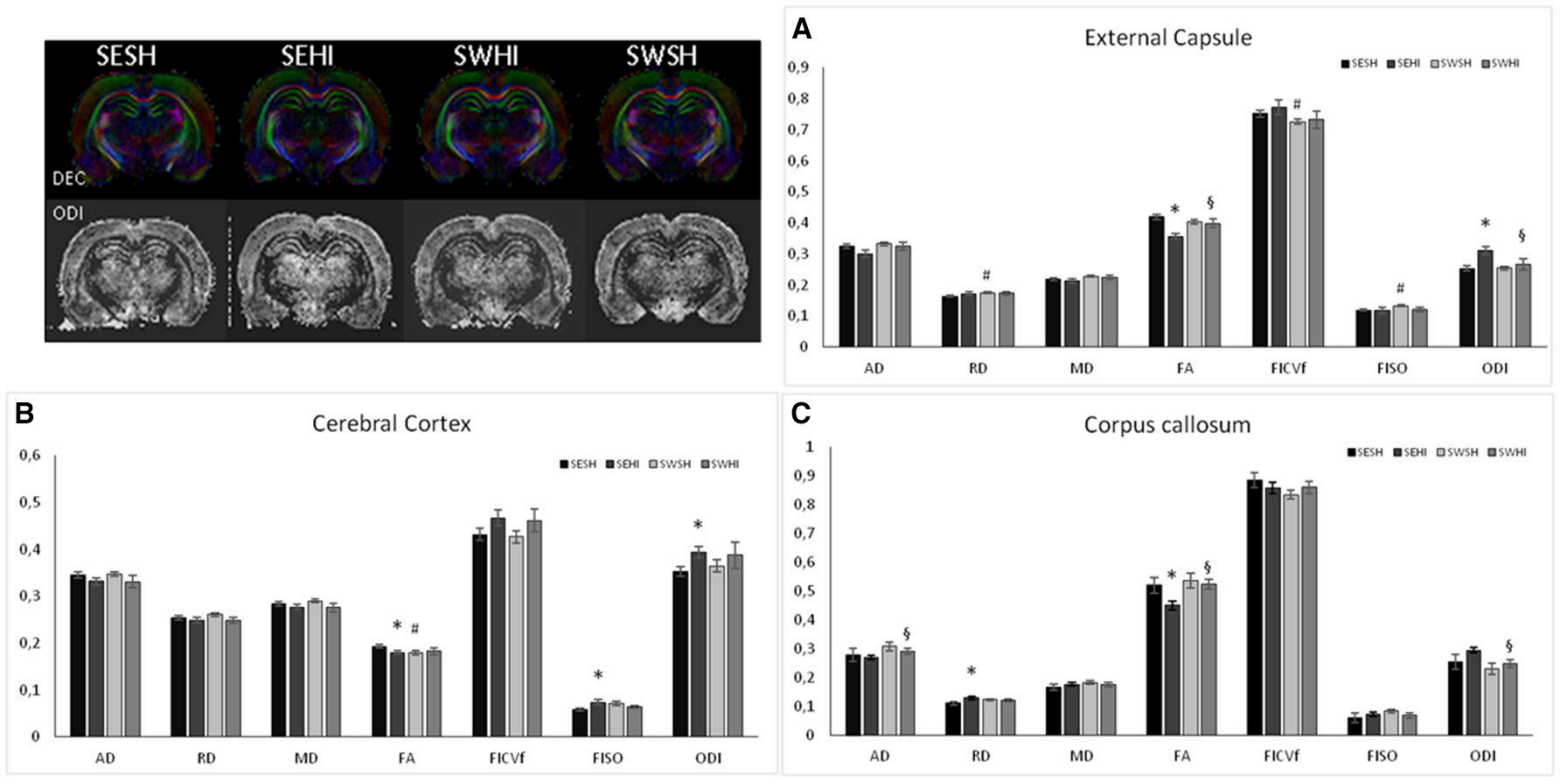
Using *ex vivo* DT-MRI long-term microstructural white matter alterations were analyzed at PND60 following neonatal HI. White matter microstructure is partially restored by pregnancy swimming. Histograms of mean values ± SEM of DTI derived parameters: Axial Diffusivity (AD), Radial Diffusivity (RD), Median diffusivity (MD), fractional anisotropy (FA) and NODDI estimates: intraneurite volume fraction (ficvf), cerebrospinal volume fraction (fiso) and orientation dispersion index (ODI) in the external capsule **(A)**, cerebral cortex **(B)**, and corpus callosum **(C)** for SESH, SEHI, SWSH and SWHI rats at P60. *SEHI vs. SESH, ^#^SESH vs. SWSH, ^§^SEHI vs. SWHI; *p* < 0.05.

At PND60, FA measurements are presented as mean values of right hemisphere cortex (C), external capsule (EC) and corpus callosum (CC) (Figures 6A–C). In the cortex, SEHI group had decreased FA (*Z* = −2.75, *p* = 0.02), and increased fiso (*Z* = −2.052, *p* = 0.04) and ODI (*Z* = −2.196, *p* = 0.028) compared to SESH, evidencing the disruption in the cortical microstructure following HI. No differences were observed in the SWHI group compared neither to SWSH nor to SESHI which implies the neuroprotection of the tissue offered by swimming.

**Figure 7:**
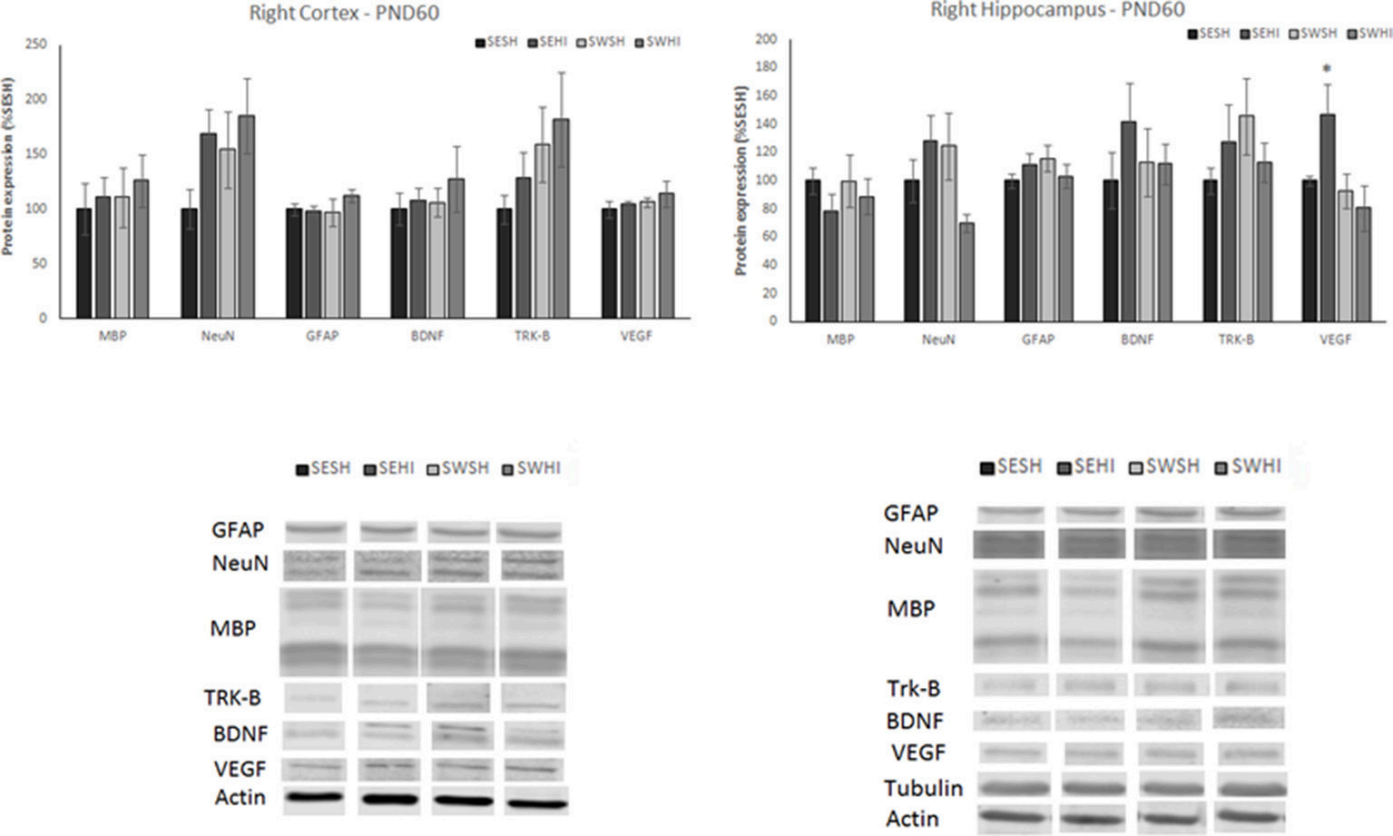
Protein expression of MBP, NeuN, GFAP, BDNF, TRK-β, and VEGF in right cortex (left upper panel) and hippocampus (right upper panel) at PND60. Lower panels: representative immunoblots of cortex (left lower) and hippocampus (right lower). WB results are plotted normalized to the SESH group level expression (100%) (mean ± SEM). Significance testing was determined using one-way ANOVA followed by Duncan's *post-hoc* using Actin or βIII-tubulin as normalizer. *SESH vs. SEHI. Significance accepted when *p* < 0.05.

In the CC, no differences between SESH and SWSH were observed. SEHI animals had increased RD (*Z* = −1.92, *p* = 0.04) and a decrease in FA (*Z* = −1.89, *p* = 0.04) compared to SESH. SWHI had increase AD (*Z* = −1.93, *p* = 0.04) and FA (*Z* = −2.47, *p* = 0.01) and decreased ODI (*Z* = −2.04, *p* = 0.01) compared to SEHI, evidencing the protective effect of swimming on myelinated structures.

In the EC, RD (*Z* = −2.92, *p* = 0.03) and f_*iso*_ (*Z* = −2.91, *p* = 0.04) were increased comparing SWSH and SESH groups. Decreased FA (*Z* = −2.82, *p* = 0.005) and increased ODI (*Z* = −2.65, *p* = 0.008) were observed comparing SEHI and SESH groups. When comparison was made between SWHI and SEHI, FA was increased (*Z* = −1.93, *p* = 0.04) and ODI decreased (*Z* = −193, *p* = 0.04) in the SWHI groups. No differences regarding microstructure were observed in the basal ganglia using NODDI derived parameters.

## Discussion

In this study, we describe the effects of a gestational swimming protocol on preventing HI-induced early metabolic damage, brain microstructure and late behavioral outcomes. Despite the extensive results presented in the literature about the benefits of maternal exercise, there are gaps in knowledge about its effects and pathways that could lead to brain protection in the offspring. We have shown that at an early stage (24 post HI), ^1^H-MRS showed that pregnancy maintained the brain energetic metabolism and limited neuronal damage. Western blotting analysis evidenced that swimming decreased the expression of proteins related to apoptotic cell death, astrogliosis and modulated neurotrophins, especially in the hippocampus. At the brain microstructural level DTI/NODDI showed that swimming caused preservation in the myelinated white matter areas. Also, gestational swimming reduced spatial memory impairments due to HI, which implies that early protection induced by swimming can confer long-term neuroprotection.

### Pregnancy swimming alters brain response to HI measured by ^1^HMRS

During hypoxia-ischemia insults there is a primary phase of energy failure (up to 24 h following injury) with a decrease in energetic brain metabolites such as ATP and PCr (58, 59), alterations in aminoacids and neurotransmitters, oxidative stress and osmoregulation failure (60). In our study, gestational swimming could limit the decrease in NAAG and total NAA (NAA+NAAG), which implies that gestational swimming can prevent neuronal damage following HI (61). Gestational swimming caused preservation of the energetic metabolism, observed by preservation of PCr concentrations. Interestingly, hypothermia, the clinical standard of HI care, has shown to increase ATP, phosphocreatine, and total NAA levels after HI (62). Also, recently it was shown that pregnancy swimming prevented the failure in the Na+/K+-ATPase caused by HI (50). The reduction of Tau and Ins in the ipsilateral cortex suggests loss of water homeostasis and alterations in glial osmolytes following HI. Both metabolites were reduced in the SEHI, and swimming could impede Ins decrease only in the SWHI group. In agreement with previous reports (16) we observed a decrease in concentration of metabolites related to cell membrane integrity (such as Mac and PE). However, swimming was not able to neither restore nor maintain the Mac and PE levels compared to SESH. The decrease in Cho observed in the SEHI group is attributed to impairments in cell membrane metabolism and to apoptosis (17, 63) and this phenomenon was reverted in the SWHI group, supporting that swimming is acting to decrease apoptosis following HIPND3. The glutamatergic neurotransmission system was altered as suggested by the decrease in the Glu and Tau, and swimming had no effect on these alterations. Contrarily to our expectations, due to the glutamine decrease, the ratio [Glu]/[Gln] was also decreased in the SWHI group, which could indicate an impairment in the Glu and Gln cycling between neurons and glia (16). However, the expression of the glutamine synthetase in the hippocampus (Figure 3D) can be interpreted as an attempt of the astrocytes to convert the excess of glutamate due to the HI into glutamine. Lac/NAA ratio reflects mitochondrial impairment and neuronal integrity and a high ratio in the first month after birth asphyxia predicts a poor 12–18 months neurodevelopmental outcome in clinical studies and has been suggested as a potential biomarker of outcome prognostic (64, 65). In our study, we observed an increase in Lac/NAA ratio in the SEHI group, prevented by gestational swimming in the SWHI. One feature of HI injury is the Lac accumulation as the consequence of the anaerobic metabolism following HI (12). We observed that the levels of Lac remained unaltered after HI, pointing to a less severe injury compared to a previous study from the group, in which this metabolite was increased 24 after injury (16). One possible interpretation for this result is that, as like most interventions that have been shown to have neuroprotective effects in HI models (62), swimming could show its effects when the lesion is not as severe, evidencing a limited recovery potential, as observed by Marcelino et al. (66).

### Pregnancy swimming decreased apoptosis and astrogliosis following HI

*In vivo* MRS can detect the disturbances caused by HI in the energy metabolism that trigger a number of pathophysiological responses that ultimately lead to different types of cell death (67, 68). HI on PND3 is well characterized as having both necrotic and apoptotic cell death (21, 23, 69). There was an increase in cleaved caspase 3 (an indicator of apoptotic cell death) in the lysate of hippocampus and cortex of SEHI 24 h after injury. Kim et al. (70) reported (in healthy animals) no difference in DG neuronal apoptotic cell death. Leite et al. (71), using hippocampal slices submitted to oxygen glucose deprivation observed a reduction in the LDH (and decreased cell death) in animals whose mothers swam during pregnancy. Maternal swimming was able to prevent this increase in the SWHI group evidencing the anti-apoptotic effects of maternal swimming as suggested in the literature (72). In agreement, pre-conditioning induced by chronic swimming is also able to protect the brain from excitotoxic events in different models (46, 73–75) as well as modulating the expression of the apoptotic effector proteins such caspase 3 in *in vivo* experiments (76). Following HIPND3, Sizonenko et al. (23) correlated the acute reduced apparent diffusion coefficient and fractional anisotropy in the ipsilateral cortex to regions of neuronal death, radial glia disruption and astrogliosis. In the present study, the SEHI group had an increase in GFAP levels (an astrogliosis index) in the cortex and the pregnancy swimming was able to minimize astroglial reaction. This is supported by the preservation in the Ins observed by ^1^HMRS. It is interesting to note the lack of data reporting the effects of exercise during pregnancy and evaluation of the astrocytes. Kim et al. (70), using a model of PVL reported a decrease in the GFAP immunoreactivity following a protocol of exercise. However, the protocol was performed in the pups, which makes the comparison more difficult.

One of the central hypothesis of the beneficial effects of gestational swimming is its ability to increase the production of neurotrophins (44, 45, 48). When evaluated 24 h following HI, we observed a decrease in BDNF in the cortex of HI groups (SE and SW). However, the receptor Trk-B was decreased only in the SEHI group. In the hippocampus, there was an increase in the neurotrophins (BDNF and VEGF) as well as in the TRK-B receptor in the SWHI group. BDNF controls the development, survival, and differentiation of the neurons through Trk-B. We can speculate that the hippocampus acts like a “sensor,” identifying the injury and increasing the production of neurotrophins. In agreement, authors observed an increase in BDNF levels in the hippocampal formation of animals whose mother swam during pregnancy (38, 46, 50). At adult age, HI increased VEGF expression in the hippocampus (related to spatial memory) of the SEHI rats but not in the SWHI (46). The increase in VEGF expression in the hippocampus during chronic epilepsy in both humans and animal models has been associated with increased angiogenic processes and blood-brain barrier disruption, which could worsen the injury (77). The expression of BDNF and the Trk-B receptor were not altered at adult age. In agreement, Marcelino et al. (66) did not find differences in the levels of BDNF at adult age and attributed this to the time point of evaluation (66). Here, we show that protein expression alteration induced by swimming in the BDNF signaling following HI seems to be more important in the early phase of injury.

### Pregnancy swimming mitigates cognitive impairments and white matter injury induced by HI

To assess the effects of pregnancy swimming over the functional impairments caused by HI at PND3, we used anxiety-related (elevated plus maze), locomotor (open field) and cognitive tests (NOR and Morris water maze). HI causes anxiety related alterations in the SEHI animals that were not observed in the SWHI animals, providing evidence of functional neuroprotection induced by gestational swimming. In agreement, Torabi et al. showed that maternal swimming prevented anxiety-related behavior in the offspring of morphine-dependent mothers (78). The motor function analysis did not reveal any gross motor deficit nor hyperactivity due to the hypoxia-ischemia model nor an improvement induced by gestational swimming. In agreement, literature has shown that hyperactivity in the open field (i.e., increased number of crossings) and in other motor tests (such as the asymmetrical use of the forelimbs in the cylinder test) using the same model are not altered by the HI PND3 which can point to the preservation of the cortico-spinal tract (50, 79). Since we did not detect a volumetric decrease in the ipsilateral hemisphere, it is reasonable to accept that the tissue injury was not sufficient to induce motor impairments. The degree of injury is highly correlated to the functional deficits and when the injury parameters are modified to obtain a more severe damage, motor alterations are observed (80–83). In agreement, Ueda et al. (81) have shown discrete motor impairments following HI attributed to a disorganization of oligodendrocyte development in layers II/III of the sensorimotor cortex (81).

At the functional level, one of the main consequences of neonatal hypoxia-ischemia is cognitive impairment, independently of the stage of brain maturation in which the injury occurs (83–85). In this context, extensive research has demonstrated that maternal exercise can potentially have positive effects on cognitive function in the offspring (45, 48, 50, 66). In our study, we did not observe non-spatial cognitive impairment (assessed in the NOR test) meaning preservation of areas in the perirhinal cortex (which plays the role of encoding information for the object discrimination performance) (86). The hippocampus, the most studied structure in the cognitive tests, seems to be highly correlated to the NOR test. Different degrees of injury (lesser than 70%) in the structure are unable to produce impairments in the test. However, MWM evidenced learning impairments in the SEHI animals, who presented greater latencies to find the platform in the last day of training and in the probe trial. Swimming had neuroprotective effect by preserving spatial memory following HI at adult age, in agreement with recent published data (50).

White matter injury is associated with a wide range of neurologic dysfunction (4, 87, 88), and can be the cause of spatial memory impairment observed in the SEHI group. Structural alterations can be observed through DTI derived parameters (median, axial and radial diffusivity and fractional anisotropy) that delineate white matter microstructural damage in animal models of perinatal brain injury in relation with altered myelination (17, 57, 89, 90). Typically, there is a reduction in MD values in the acute phase of ischemia, and in FA values in the subacute/chronic phase (91). In our study, in the early phase after injury (24 h post HI) we did not detect any substantial differences in the DTI derived parameters observed in the cortex as well as in the external capsule. However, at PND60 ipsilateral hemisphere of SEHI animals showed a decrease in FA in the assessed structures (cortex, external capsule and corpus callosum) not observed in SWHI groups. Also, ODI was increased in SEHI groups in cerebral cortex and external capsule. Such alterations were partially recovered in the SWHI group, providing evidence that the myelination long-term impairment after HI injury was partially protected by pregnancy swimming.

We demonstrate that exercise during pregnancy is able to modulate brain functioning and to adapt its metabolism in order to protect itself against HI-induced damage. This adaptation induced the inhibition of apoptotic cell death, astrogliosis and the preservation of the white matter structure, reducing behavioral outcomes. To define the relationship of diffuse white matter injury sparing, further analysis of the cell types and of the damage and repair mechanisms involved will be necessary. The findings of this work indicate that maternal swimming modulates several pathways related to the HI cascade, denoting that gestational interventions have the potential to induce long-term neuroprotective effects on biomarkers and should be examined in future human studies.

## Author contributions

ES: conception of the study, acquisition, analysis and interpretation of data, drafting the article. YVdeL: acquisiton, analysis and interpretation of data, drafting the article. AdS, JR, and AT: acquisition of data. SS: supervisor, conception, data analysis, critical revision of the article, final approval.

### Conflict of interest statement

The authors declare that the research was conducted in the absence of any commercial or financial relationships that could be construed as a potential conflict of interest.
